# Supplementary material for: HIVprotI: an integrated web based platform for prediction and design of HIV proteins inhibitors
Source: J Cheminform. 2018 Mar 9;10:12. doi: 10.1186/s13321-018-0266-y (PMC5845081; doi:10.1186/s13321-018-0266-y)
Supplement: Supplementary file 2 — Additional file 2. Source code of HIVProtI web server. [file 13321_2018_266_MOESM2_ESM.zip › HIVprotI_Source-code/hivprotI/weka-3-6-6/doc/weka/gui/boundaryvisualizer/package-summary.html]

weka.gui.boundaryvisualizer


---


|  |  |  |
| --- | --- | --- |
| **Overview** \| **Package** \| Class \| **Tree** \| **Deprecated** \| **Index** \| **Help** \| **Weka's home** | |  |
| **PREV PACKAGE**   **NEXT PACKAGE** | **FRAMES**    **NO FRAMES**     **All Classes** |  |


---

## Package weka.gui.boundaryvisualizer

| **Interface Summary** | |
| --- | --- |
| **DataGenerator** | Interface to something that can generate new instances based on a set of input instances |

| **Class Summary** | |
| --- | --- |
| **BoundaryPanel** | BoundaryPanel. |
| **BoundaryPanelDistributed** | This class extends BoundaryPanel with code for distributing the processing necessary to create a visualization among a list of remote machines. |
| **BoundaryVisualizer** | BoundaryVisualizer. |
| **KDDataGenerator** | KDDataGenerator. |
| **Messages** | Messages. |
| **RemoteBoundaryVisualizerSubTask** | Class that encapsulates a sub task for distributed boundary visualization. |
| **RemoteResult** | Class that encapsulates a result (and progress info) for part of a distributed boundary visualization. |

---


|  |  |  |
| --- | --- | --- |
| **Overview** \| **Package** \| Class \| **Tree** \| **Deprecated** \| **Index** \| **Help** \| **Weka's home** | |  |
| **PREV PACKAGE**   **NEXT PACKAGE** | **FRAMES**    **NO FRAMES**     **All Classes** |  |


---
